# Supplementary material for: Electrophysiological decoding captures the temporal trajectory of face categorization in infants
Source: Dev Cogn Neurosci. 2025 Aug 7;75:101601. doi: 10.1016/j.dcn.2025.101601 (PMC12362079; doi:10.1016/j.dcn.2025.101601)
Supplement: Supplementary file 1 — Supplementary material [file mmc1.pdf]

– Supplementary Online Material –

Electrophysiological decoding captures the temporal trajectory of face categorization in infants

Roman Kessler[1] and Michael A. Skeide[1] ✉

[1] Research Group Learning in Early Childhood,

Max Planck Institute for Human Cognitive and Brain Sciences,

Stephanstraße 1A, 04103 Leipzig, Germany

✉ e-mail: skeide@cbs.mpg.de

Correspondence should be addressed to Michael A. Skeide

Table S1 | Demographics and trials counts per participant. Number of trials in each of the four stimulus conditions (human 1, human 2, monkey 1, and monkey 2) after quality control. Age refers either to the age in months at the experimental session, if only one session was included, or to the average age across sessions, if two sessions were included. Sex: m=male, f=female.

Participant	Number of trials per condition	Age (months)	Sex
**sub-001**	126	13	m
**sub-002**	108	8	f
**sub-003**	105	9	f
**sub-004**	135	9	m
**sub-005**	51	9	m
**sub-006**	82	7.5	m
**sub-007**	71	13	m
**sub-008**	84	7.5	m
**sub-009**	105	8.5	f
**sub-010**	113	7.5	f
**sub-012**	144	9	f
**sub-013**	115	9.5	f
**sub-014**	73	9	f
**sub-015**	139	9	f
**sub-016**	38	8	m
**sub-017**	98	7.5	f
**sub-019**	76	9	f

**sub-020**	109	8.5	m
**sub-021**	135	8.5	f
**sub-022**	150	7.5	f
**sub-023**	98	7	m
**sub-024**	96	8	m
**sub-025**	56	6	m
**sub-026**	76	7.5	m
**sub-028**	90	7	m
**sub-030**	159	8	m
**sub-032**	65	8.5	m
**sub-033**	86	8	f
**sub-034**	75	8.5	f
**sub-035**	66	8.5	m
**sub-036**	93	8	f
**sub-038**	121	8.5	f
**sub-039**	125	7	m
**sub-040**	48	5	f
**sub-041**	76	8	m
**sub-042**	99	8	f
**sub-043**	156	8	f
**sub-044**	93	8.5	m
